# Brazos Valley Medical Association

**Published:** 1903-10

**Authors:** W. W. Greer, W. B. Briggs

**Affiliations:** President; Easterly, Texas; Secretary; Easterly, Texas


### Society Notes.

### Brazos Valley Medical Association.

EASTERLY, TEXAS, September 9, 1903.

DEAR DOCTOR: The next meeting of the Brazos Valley Medical Association will be held in Hearne on the 10th and 11th of November, 1903.

You are very earnestly requested to prepare a paper on some theme of your own selection. You will have ample time, but commence now. Please do not fail to write a paper, and send the subject you select to Dr. W. B. Briggs, Easterly, Texas, at once.

As much business of importance will be transacted, a large attendance is desired.

W. W. GREER, President.

W. B. BRIGGS, Secretary.

